# Genetic markers associated with ferroptosis in Alzheimer’s disease

**DOI:** 10.3389/fnagi.2024.1364605

**Published:** 2024-04-22

**Authors:** Yuting Sun, Yu Xiao, Qin Tang, Wei Chen, Lu Lin

**Affiliations:** ^1^Department of Clinical Laboratory, The Fourth People’s Hospital of Chengdu, Chengdu, China; ^2^Department of Clinical Laboratory, The Clinical Hospital of Chengdu Brain Science Institute, MOE Key Lab for Neuroinformation, University of Electronic Science and Technology of China, Chengdu, China; ^3^Psychosomatic Medicine Center, The Fourth People’s Hospital of Chengdu, Chengdu, China; ^4^Psychosomatic Medicine Center, The Clinical Hospital of Chengdu Brain Science Institute, MOE Key Lab for Neuroinformation, University of Electronic Science and Technology of China, Chengdu, China

**Keywords:** Alzheimer’s disease, ferroptosis, immune cell infiltration, diagnosis, ceRNA

## Abstract

**Objective:**

Ferroptosis is implicated in the pathogenesis of neurodegenerative disorders such as Alzheimer’s disease, Parkinson’s disease, and vascular dementia, implying that it may have a regulatory effect on the progression of these diseases. However, the specific role of ferroptosis-related genes (FRGs) in Alzheimer’s disease (AD) is not yet fully understood. The aim of the study was to detect ferroptosis related genes with regulatory functions in the disease and explore potential mechanisms in AD.

**Methods:**

Hub FRGs were obtained through multiple algorithms based on the GSE5281 dataset. The screening process was implemented by R packages including limma, WGCNA, glm and SVM-RFE. Gene Ontology classification and pathway enrichment analysis were performed based on FRGs. Biological processes involved with hub FRGs were investigated through GSVA and GSEA methods. Immune infiltration analysis was performed by the R package CIBERSORT. Receiver operating characteristic curve (ROC) was utilized to validate the accuracy of hub FRGs. The CeRNA network attempted to find non-coding RNA transcripts which may play a role in disease progression.

**Results:**

DDIT4, MUC1, KLHL24, CD44, and RB1 were identified as hub FRGs. As later revealed by enrichment analysis, the hub FRGs had important effects on AD through involvement in diverse AD pathogenesis-related pathways such as autophagy and glutathione metabolism. The immune microenvironment in AD shows increased numbers of resting NK cells, macrophages, and mast cells, with decreased levels of CD8 T cells when compared to healthy samples. Regulatory T cells were positively correlated with MUC1, KLHL24, and DDIT4 expression, while RB1 showed negative correlations with eosinophils and CD8 T cells, suggesting potential roles in modulating the immune environment in AD.

**Conclusion:**

Our research has identified five hub FRGs in AD. We concluded that ferroptosis may be involved in the disease.

## 1 Introduction

Alzheimer’s disease (AD) is a neurodegenerative disorder that affects the cerebral cortex and commonly occurs in older adults (Weller and Budson, 2018; Scheltens et al., 2021). Due to the aging population, the incidence of AD is increasing, making it a significant age-related health concern. AD typically presents with progressive memory loss, cognitive impairment, and motor dysfunction. Its underlying causes and mechanisms remain largely unknown, posing significant challenges for its prevention and treatment (Blennow and Zetterberg, 2018). The pathogenesis of AD is a complex multifactorial process that involves the interaction of several aspects such as abnormal protein deposition, neuronal damage, inflammatory response, and oxidative stress. The presence of beta-amyloid (Aβ) plaques in the brains of AD patients is one of the key features of the disease. These plaques interfere with signaling between neurons, leading to impaired neuronal function and death. Another important pathological feature is neurofibrillary tangle (NFTs), caused mainly by abnormal aggregation of Tau proteins. These tangles lead to structural damage to neurons and affect normal neuronal function. Inflammatory responses and oxidative stress also play an important role in the pathogenesis of AD. The inflammatory response can accelerate neuronal damage, while oxidative stress leads to cell membrane and DNA damage, exacerbating disease progression.

Ferroptosis is a novel form of iron-dependent cell death that differs from traditional cell death such as apoptosis and necrosis (Dixon et al., 2012). This process involves a complex regulatory network. Ferroptosis is characterized by the accumulation of intracellular iron leading to increased oxidative stress, which ultimately triggers lipid peroxidation and cell death (Cao and Dixon, 2016). Cellular scavenging of lipid peroxides relies primarily on the action of glutathione peroxidase 4 (GPX4), a glutathione peroxidase that is an important antioxidant enzyme within the cell. Cells take up cystine from outside the cell via the cystine/glutamate antiporter (System Xc-), and cystine is an important raw material for the intracellular biosynthesis of the reducing substance glutathione (GSH). GPX4 can protect cell membranes from oxidative damage by using glutathione as a substrate to reduce lipid peroxides to normal phospholipid molecules. When System Xc- is inhibited, GSH is depleted, ultimately leading to inactivation of the GPX4, resulting in the accumulation of lipid peroxidation and ferroptosis (Wei et al., 2020). During the ferroptosis, mitochondria in ferroptosis show smaller size, wrinkled membranes, reduced or absent cristae, and fragmented outer membranes. Nuclear morphology changes are not prominent. Additionally, there is an association between ferroptosis and neurodegenerative diseases such as Alzheimer’s disease, Huntington’s disease and Parkinson’s syndrome (Cardoso et al., 2017).

It has been shown that GSH/GPX4 is involved in ferroptosis in AD. Decreased GSH levels in the hippocampus and frontal cortex are associated with severe cognitive impairment, suggesting that GSH may be a biomarker for AD (Ayton et al., 2020). However, oral GSH supplementation is difficult to restore brain GSH levels because GSH is easily hydrolyzed and difficult to cross the blood–brain barrier. N-acetyl-L-cysteine (NAC) can efficiently cross the blood–brain barrier to enter the brain. In an animal model of AD, NAC modulates brain GSH levels to exert an anti-lipid peroxidative effect. The use of NAC can enhance the permeability of cell membrane and mitochondrial membrane, thereby increasing the level of GSH, inhibiting ferroptosis and exerting neuroprotective effects. Furthermore, ferroptosis interacts with Aβ and amyloid precursor protein (APP) (Tardiolo et al., 2018; Terluk et al., 2019). Aβ reduces Fe^3+^ to Fe^2+^, leading to the generation of free radicals and damage to neurons. APP is a precursor of Aβ. If cleaved by β-secretase, it produces neurotoxic Aβ. Furin regulates the activity of secretase. Iron overload inhibits the expression of Furin and enhances the activity of β-secretase, which increases Aβ production (Ward et al., 2014). In addition, ferroptosis interacts with tau proteins and NFTs. Dysregulation of brain iron homeostasis is closely related to tau proteins and NFTs. Tau proteins are hyperphosphorylated and aggregated into NFTs. Iron regulates tau protein phosphorylation, which affects iron efflux. This leads to the vicious cycle of neuronal iron deposition and formation of NFTs (Wang and Mandelkow, 2016). Given the interplay between ferroptosis and AD, exploring potential links between the two might identify novel therapeutic targets for treatment. The objective of this work was to explore whether ferroptosis-related genes could play a key role in AD and analyze their effects on the immune microenvironment and pathways using bioinformatics.

## 2 Materials and methods

### 2.1 Data acquisition and processing

GSE5281 was retrieved from the Gene Expression Omnibus (GEO) database.^1^ The GSE5281 dataset embodied a total of 161 samples, including 74 healthy samples and 87 AD samples. This dataset was considered as a training set for analysis by the main body of this research. The limma R package was used to remove sample differences. The FRGs (*n* = 259) used in this study were obtained from FerrDb.^2^

### 2.2 Differential expression analysis

The GSE5281 series matrix files were annotated with an official gene symbol using the data table from the microarray platform, and then gene expression matrix files were obtained. FactoMineR and factoextra R packages were used to analyze and draw the plot. Principal component analysis (PCA) was conducted using the whole gene list. Limma R package was used to remove batch effects and conduct DEG analysis. The threshold of DEGs was set as |log2(fold-change)| > 1 and *p* < 0.05. GO function and KEGG pathway enrichment analyses were conducted to explore the core mechanism and pathway of genes obtained through the above process. R package clusterProfiler was used to conduct GO and KEGG pathway enrichment analysis. The R package ggplot2 was adopted to visualize the results of functional enrichment analysis. Statistical significance was set at adjusted-*p* < 0.05.

### 2.3 Co-expression network construction by WGCNA

The “WGCNA” R package was used to construct a co-expression network for all genes in AD and healthy brain tissue samples. Genes with the top 25% mean were filtered by the algorithm for further analysis. First, the hclust function was utilized for sample clustering to eliminate outlier samples, with the parameter “method = average” set for distance calculation. GSM119676 was deleted to ensure that the results of the network construction were reliable. 73 AD samples and 87 healthy samples were involved in WGCNA analysis. Then, the number 7 was selected as the soft threshold for network construction by function pickSoftThreshold. The gene network was constructed by the one-step method. Second, after transforming the adjacency matrix into a topological overlap matrix (TOM), a hierarchical cluster tree of genes was generated by hierarchical clustering. The identification of highly correlated co-expressed gene modules was made by the dynamic tree cut method, and the connection between the module eigengene (ME) and disease was analyzed using the Pearson correlation coefficient. Finally, yellow module genes were derived as DRGs for further analysis.

### 2.4 Identification of hub FRGs for AD

The least absolute shrinkage and selection operator (LASSO) algorithm was applied with the glmnet package to reduce the dimensions of the data. The DRGs and DEGs between AD patients and healthy samples were identified with the LASSO algorithms. Response type was set as binomial and alpha was set as one. Meanwhile, a support vector machine-recursive feature elimination (SVM-RFE) model was established with a SVM package, which was compared by the average misjudgment rates of their 10-fold cross-validations. Finally, hub ferroptosis-related genes for AD were identified by overlapping genes derived from the two algorithms. Venn diagrams were drawn using the jvenn plug-in online.^3^ To evaluate the diagnostic performance of each selected gene, we calculated the receiver operating characteristic (ROC) curve and determined the corresponding area under the curve (AUC) accuracy, sensitivity, and specificity. This process was implemented through R package glm.

### 2.5 Single-gene gene set enrichment analysis enrichment analysis

This analysis is implemented in the GSEA package in R. To further explore the related pathways of the five hub ferroptosis-related genes, we calculated the correlation between the hub ferroptosis-related genes and all other genes in the GSE5281 dataset. Subsequently, all genes were sorted according to their correlations from high to low, and these sorted genes were considered to be the gene set to be tested. Meanwhile, the KEGG signaling pathway set was invoked as a predefined set to detect its enrichment in the gene set. A *p*-value of less than 0.05 was deemed statistically significant.

### 2.6 Single-gene gene set variation analysis enrichment analysis

GSVA is a gene set variation analysis. This analysis was implemented in the GSVA package in R. In this study, we utilized the median of hub FRGs expression and divided them into high- and low-expression groups. Then, we used the KEGG pathway set as the background gene set to perform GSVA analysis on each hub of ferroptosis-related genes. Simultaneously, we applied the limma package to analyze the difference in GSVA scores of the hub FRGs’ high-expression group samples. The difference screening condition was *t* > 0, *p* < 0.05. We demonstrated the top 10 GSVA-KEGG pathways through a visual plot.

### 2.7 Immune infiltration analysis

The proportion of 22 types of infiltrating immune cell types in each tissue from the GSE5281 dataset was predicted using the R package CIBERSORT. The correlation between hub FRGs and immune cells was calculated using the R package GSVA. The correlation plot was displayed using the R package pheamap.

### 2.8 Construction of ceRNA network

The relationship between mRNAs and miRNAs was predicted on the basis of four databases: MirGeneDB2.1, miRDB, miRBase, and miRWalk. We selected the miRNAs which appeared in more than two databases. The relationship between miRNAs and lncRNAs was predicted on the basis of the ENCORI database. Subsequently, we integrated the interaction between mRNAs, miRNAs and lncRNAs to construct a ceRNA regulatory network. Cytoscape (version 3.10.1) was used to visualize the ceRNA network.

### 2.9 Statistical analysis

All data processing and analyses were done using R software (version 4.3.0). To compare the two groups of continuous variables, the statistical significance of normally distributed variables was assessed using an independent Student’s *t*-test. For non-normally distributed variables, differences were analyzed using the Mann–Whitney U test (Wilcoxon rank sum test). The Chi-square test or Fisher’s exact test was utilized to compare and evaluate the statistical differences between the two groups of categorical variables. All *p*-values were two-tailed, and a threshold of *p* < 0.05 was considered statistically significant.

## 3 Results

### 3.1 Identification of DEGs in GSE5281

**Figure 1:**
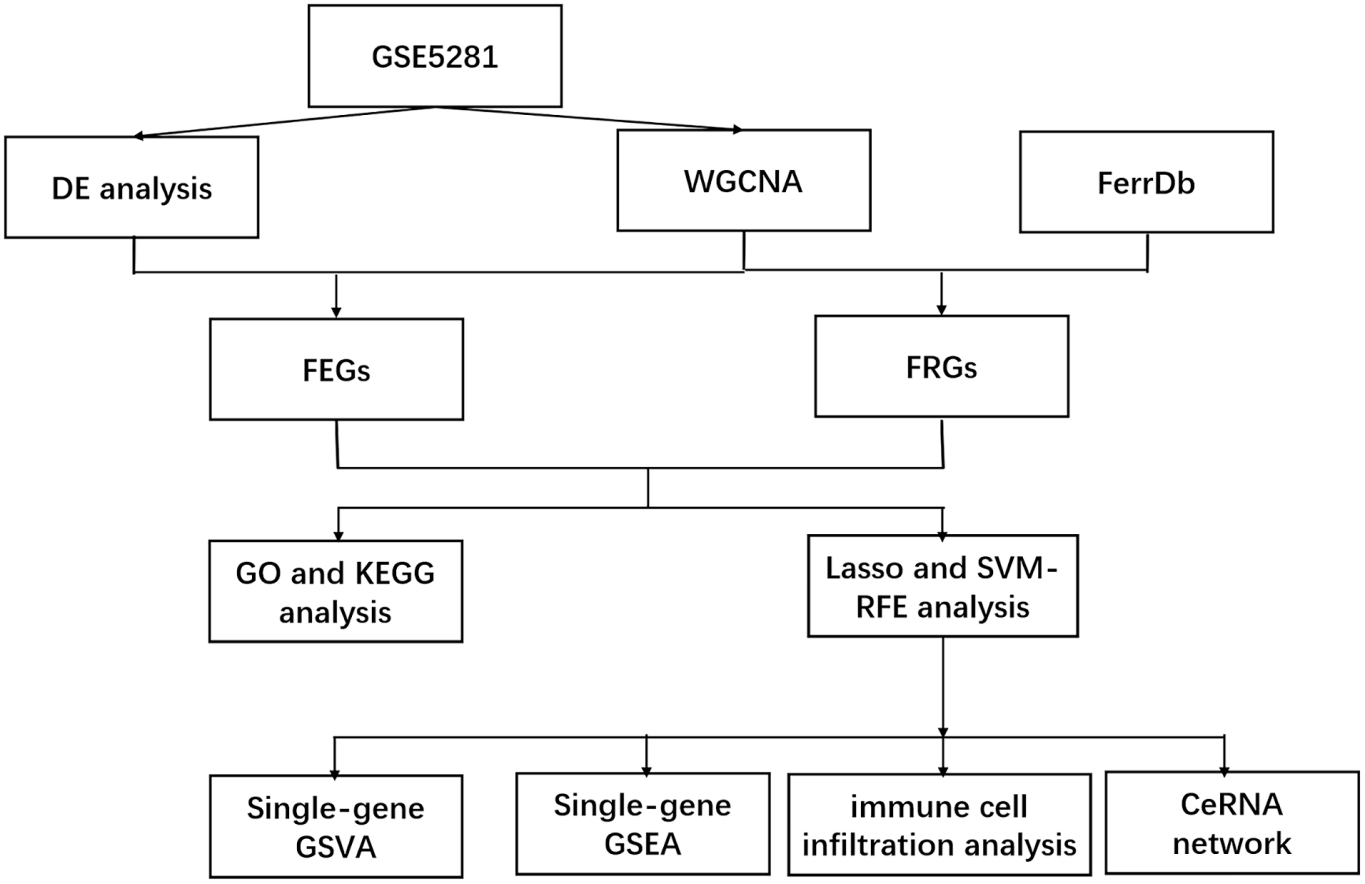
Work flow.

**Figure 2:**
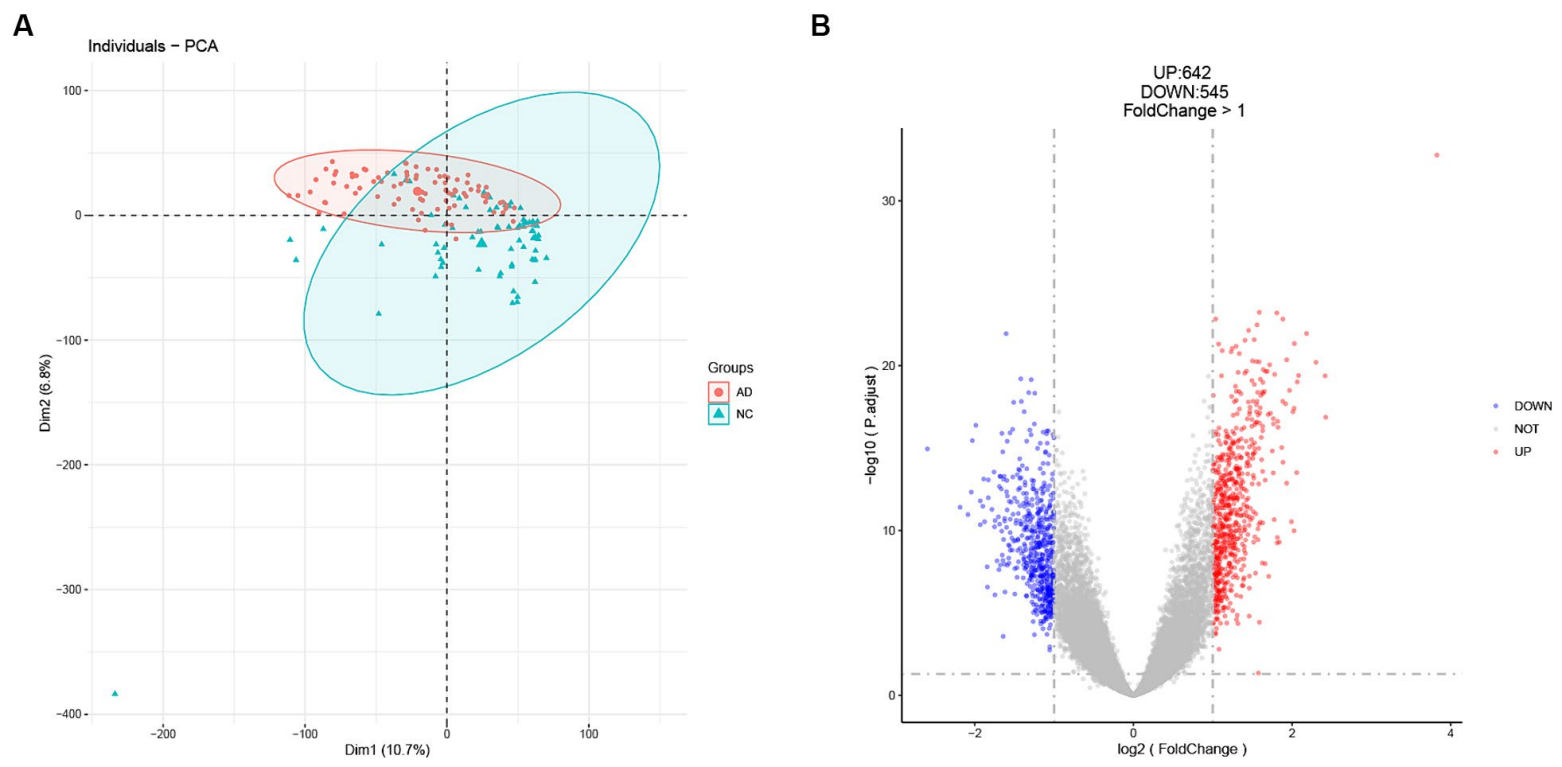
Identification of DEGs in the GSE5281 dataset. **(A)** Principal component analysis (PCA) shows a significant difference between healthy and AD groups. **(B)** Volcano plot for DEGs analysis between healthy and AD groups.

The workflow of this study is shown in Figure 1. In our analysis of the GSE5281 dataset, we utilized principal component analysis (PCA) to reduce the dimensions of the expression profiles and assess the variability in sample grouping. The result showed that the first principal component (Dim1) accounted for 10.7% of the total variance. The second principal component (Dim 2) accounted for an additional 6.8% of the total variance (Figure 2A). The PCA plot showed that the variation between the AD and healthy groups was comparatively significant. Furthermore, we identified a total of 1,187 differentially expressed genes (DEGs) in the GSE5281 dataset through the Limma R package. Among these DEGs, 642 were found to be upregulated, while 545 were downregulated. To visualize the distribution of these DEGs, particularly in relation to Alzheimer’s disease (AD), we depicted a volcano plot in Figure 2B.

### 3.2 WGCNA of GSE5281

**Figure 3:**
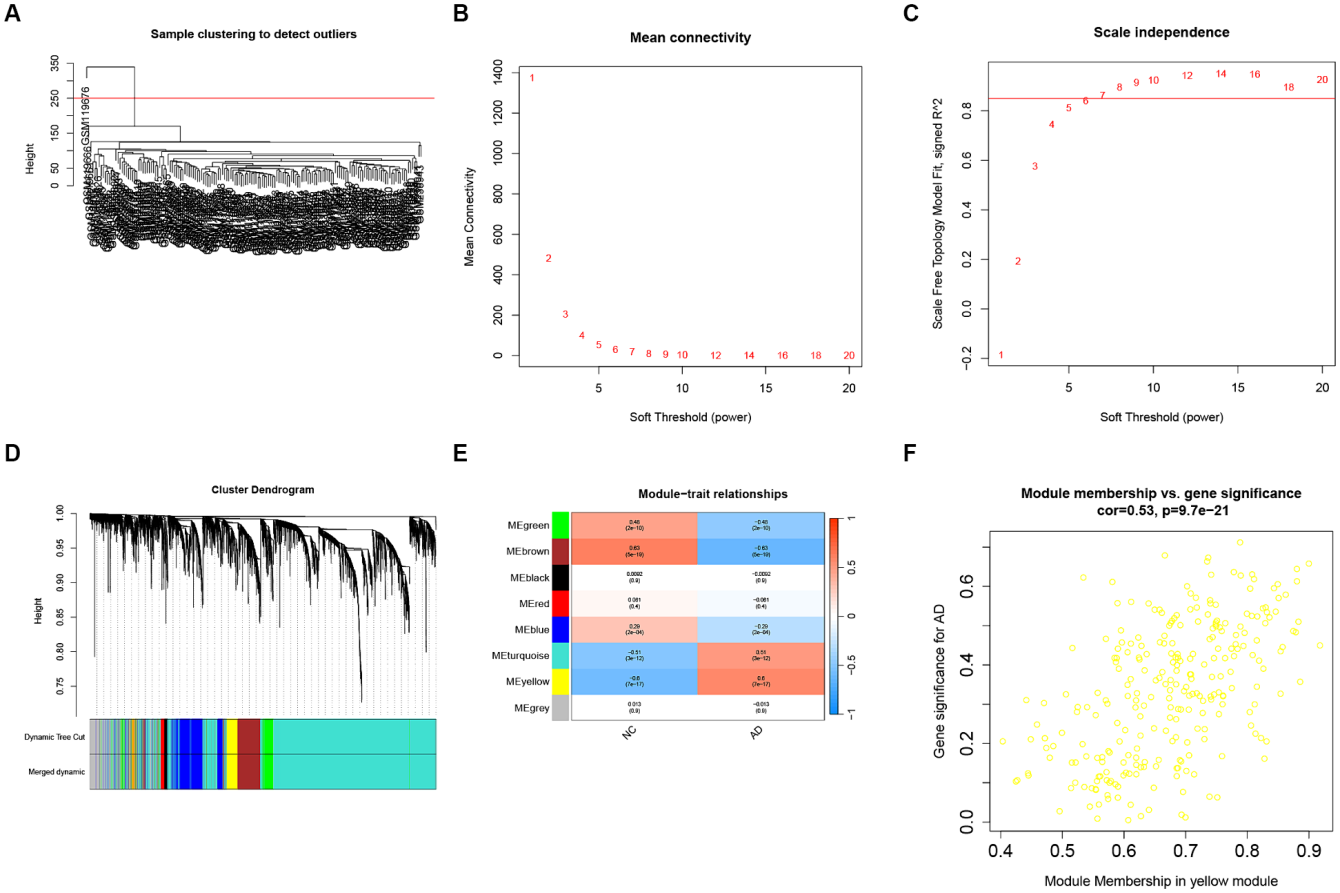
WGCNA analysis for disease-related genes (DRGs). **(A)** Samples clustering to detect the outliers. **(B)** The corresponding scale-free topological model fit indices at different soft threshold powers. **(C)** The corresponding mean connectivity values at different soft threshold powers. **(D)** Cluster dendrogram of genes. **(E)** Correlations between different modules and clinical traits. **(F)** Correlation of module membership and gene significance in the yellow module.

We conducted a WGCNA analysis on the GSE5281 dataset to explore the disease-related genes (DRGs). For sample quality control, we performed sample clustering and identified an outlier, GSM119676, which was subsequently removed (Figure 3A). The WGCNA analysis included 87 AD samples and 73 healthy human samples, comprising a total of 23,521 genes. We selected the top 5,880 genes based on their mean values for WGCNA analysis in this study. In order to achieve scale-free networks, we set the threshold as *R*^2^ = 0.85. The optimal soft-threshold power of 7 was selected (Figures 3B,C) to construct hierarchical clustering trees using the WGCNA R packages (Figure 3D). Subsequently, we constructed co-expression networks to investigate the associations between clinical features and these modules (Figure 3E). Analysis of the results revealed that the yellow module, containing 267 genes, showed a strong positive correlation with AD (Cor = 0.53, *p* = 9.7e^−21^) (Figure 3F).

### 3.3 Ferroptosis-related genes identification in AD

**Figure 4:**
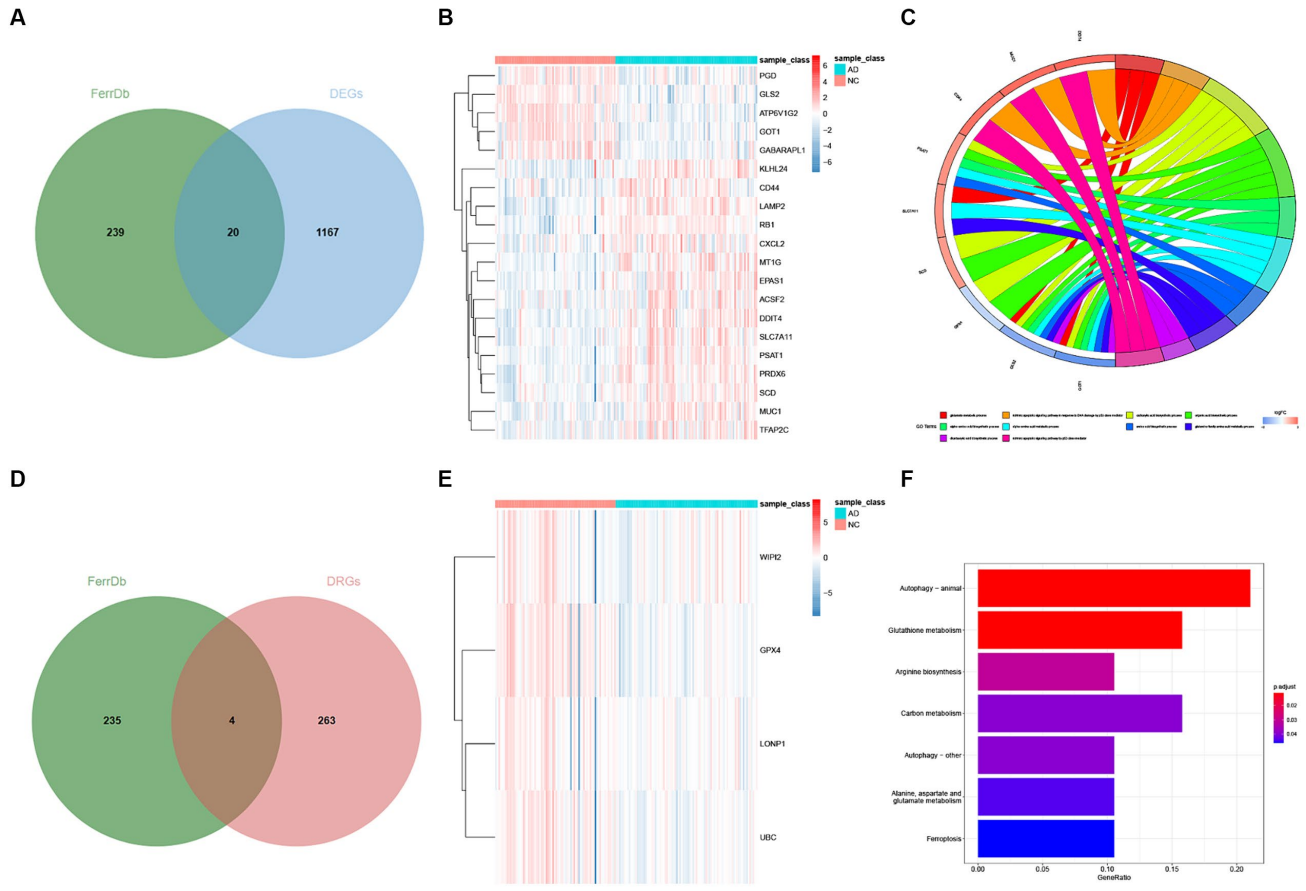
Identification of DE-FGs and DR-FGs. **(A)** Venn diagram for DE-FGs in DEGs and FerrDb **(B)** Heatmap plot for DE-FGs in FerrDb and DEGs **(C)** GO enrichment analysis for FRGs **(D)** Venn diagram for DR-FGs in DRGs and FerrDb **(E)** Heatmap plot for DR-FGs in DRGs and FerrDb **(F)** KEGG enrichment analysis for FRGs.

**Figure 5:**
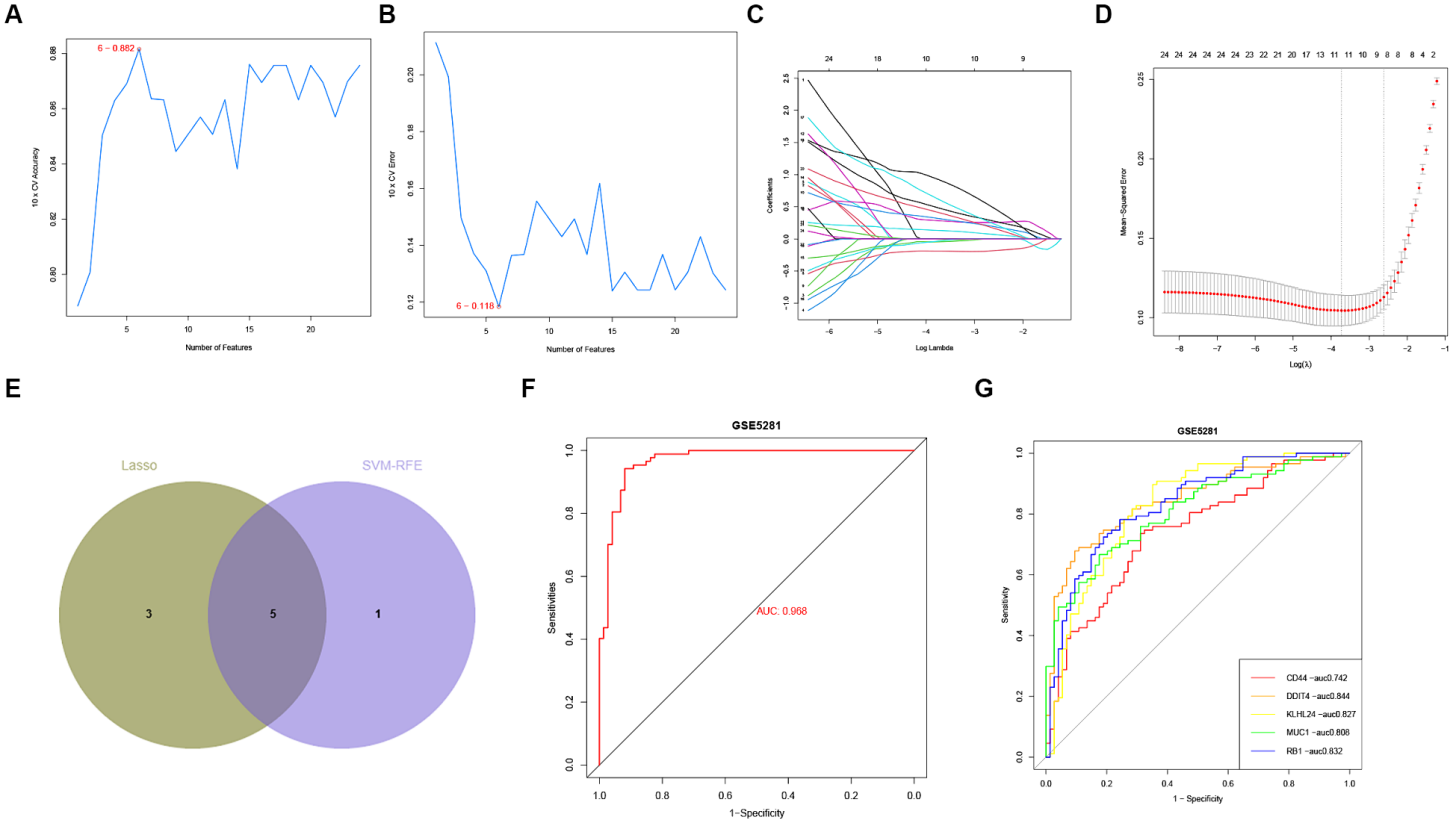
Identification of hub FRGs. **(A,B)** SVM-RFE algorithm to identify the optimal combination of feature genes. Finally, 6 genes (maximal accuracy = 0.882, minimal RMSE = 0.118) were identified as the optimal feature genes. **(C,D)** By LASSO logistic regression algorithm, with penalty parameter tuning conducted by 10-fold cross-validation, was used to select 24 FRGs. **(E)** The 5 hub FRGs obtained from the LASSO and SVM-RFE models. **(F)** Logistic regression model to identify the AUC of disease samples. **(G)** ROC curves for each hub FRGs.

We first obtained ferroptosis-related genes by overlapping the differentially expressed genes (DEGs) and (disease-related genes) DRGs with the FerrDb database separately (Figures 4A,D). To visually represent the expression differences, we generated a heatmap for the DE-FRGs and the DR-FRGs (Figures 4B,E). In total, we obtained 24 ferroptosis-related genes (FRGs) that consisted of both the DE-FRGs and DR-FRGs. A total of 24 ferroptosis-related genes (FRGs) are shown in Supplementary Table S4. To gain further insights into the potential functions of these FRGs at the biological level, we performed GO and KEGG enrichment analyses. The GO enrichment analysis revealed that the FRGs were significantly enriched in 39 biological processes (BPs) and 9 molecular functions (MFs). Notably, the most prominent projects included the glutamate metabolic process, intrinsic apoptotic signaling pathway in response to DNA damage by p53 class mediator, carboxylic acid biosynthetic process, organic acid biosynthetic process, and six other GO terms (Figure 4C). Furthermore, the KEGG enrichment analysis showed that the FRGs were involved in various pathways related to ferroptosis and other biological processes (Figure 4F). These pathways included glutathione metabolism, cysteine and methionine metabolism, p53 signaling pathway, and several other KEGG pathways. In summary, through overlapping the DEGs and DRGs with FerrDb, we obtained a set of FRGs that were further analyzed for their biological functions using GO and KEGG enrichment analyses. This analysis revealed their involvement in numerous biological processes and pathways related to ferroptosis and other cellular functions (See Figure 5).

### 3.4 Hub FRGs were identified for AD

**Figure 6:**
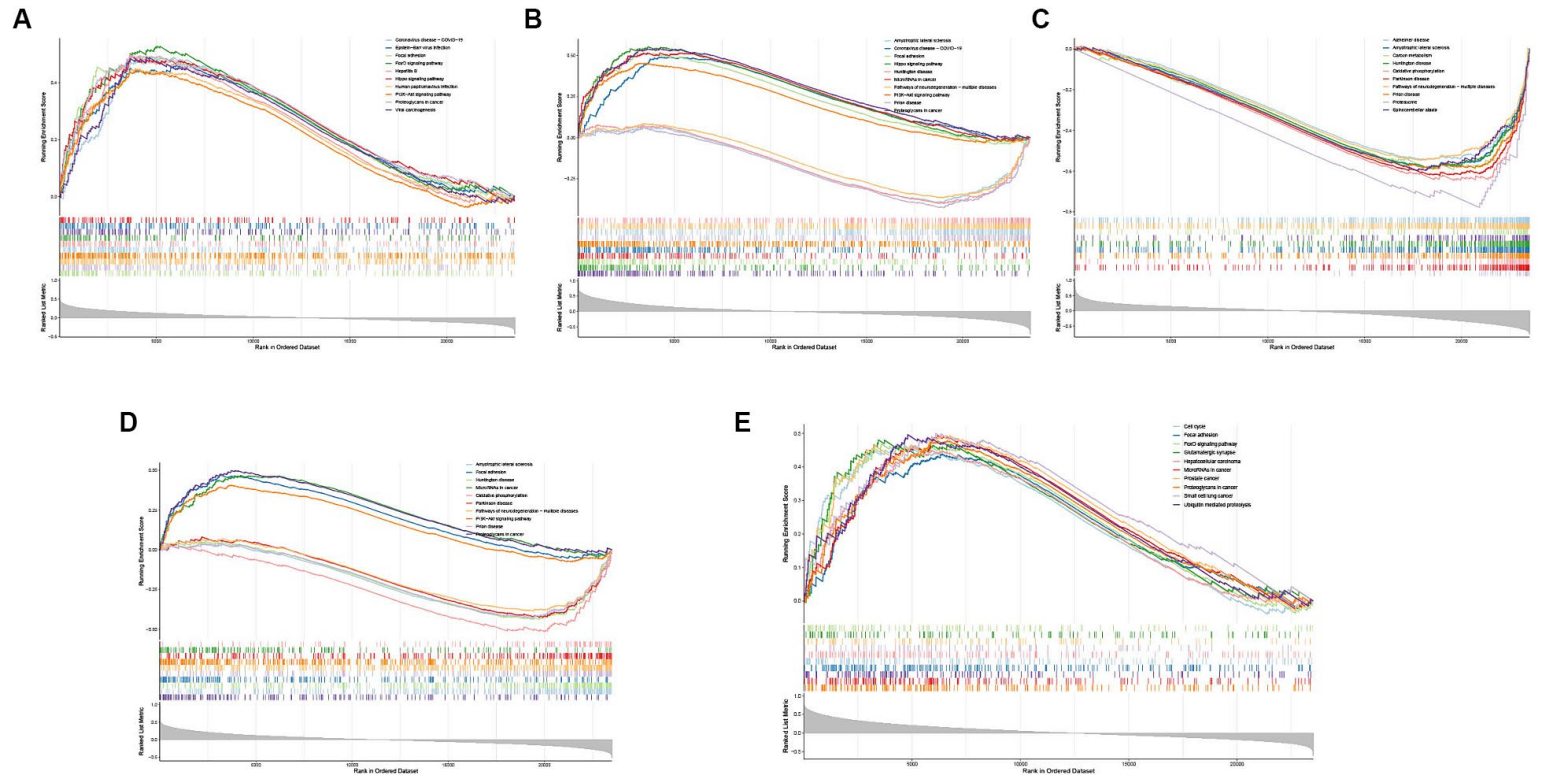
Single-gene GSEA-KEGG pathway analysis in CD44 **(A)**, DDIT4 **(B)**, KLHL24 **(C)**, MUC1 **(D)**, RB1 **(E)**.

In order to assess the regulatory potential of the ferroptosis-related genes (FRGs) in Alzheimer’s disease (AD), we applied two machine learning algorithms (LASSO and SVM-RFE) to the GSE5281 dataset to identify hub FRGs that could distinguish AD patients from healthy individuals. First, we utilized the LASSO logistic regression algorithm with penalty parameter tuning conducted by 10-fold cross-validation. This analysis narrowed down the FRGs to a set of AD-related features (Figures 6A,B). Next, we applied the SVM-RFE algorithm to further filter the initial set of 24 FRGs and identify the optimal combination of featured genes. Through this process, we identified 6 genes (with a maximal accuracy of 0.882 and minimal RMSE of 0.118) as optimally featured genes (Figures 6C,D). The results obtained from the LASSO and SVM-RFE models were then intersected to identify the overlap of key FRGs. In this analysis, 5 hub FRGs (DDIT4, MUC1, KLHL24, CD44, and RB1) were identified as the most relevant genes for distinguishing AD from healthy individuals (Figure 6E). These 5 hub FRGs can serve as potential biomarkers or therapeutic targets for further analysis and investigation in the context of AD (Supplementary Table S1). We utilized a logistic regression model based on the 5 hub FRGs mentioned above. The resulting ROC curves demonstrated that our logistic regression model, incorporating five hub FRGs, exhibited a high discriminatory ability between healthy and AD samples, with an AUC of 0.968 (Figure 6F). Furthermore, to assess the individual gene performance in distinguishing AD from healthy samples, we generated ROC curves for each of the five hub FRGs. Figure 6G illustrates that all genes achieved an AUC greater than 0.6. Collectively, these findings indicate that the logistic regression model outperforms individual marker genes in terms of accuracy and specificity when distinguishing AD samples from healthy samples.

### 3.5 GSVA and GSEA analysis of hub FRGs

**Figure 7:**
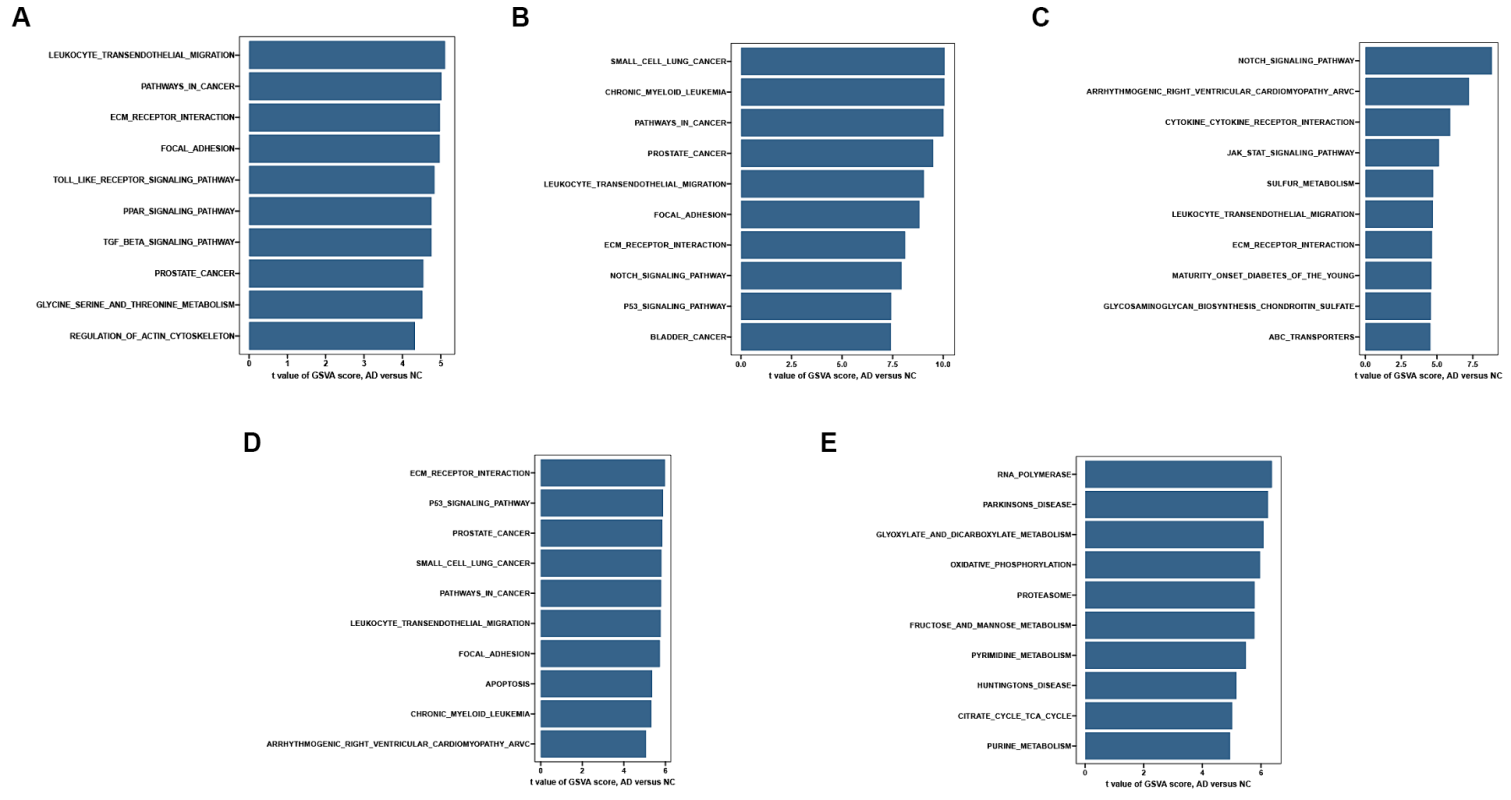
Single-gene GSVA-KEGG pathway analysis in CD44 **(A)**, DDIT4 **(B)**, KLHL24 **(C)**, MUC1 **(D)**, RB1 **(E)**.

To explore the potential functions of hub FRGs, we conducted a single-gene GSEA-KEGG pathway analysis. The top 10 pathways enriched for each marker gene were illustrated in Figures 6A–E. After a comprehensive analysis, we found that hub FRGs were enriched in neurodegenerative disorders including Amyotrophic lateral sclerosis, Huntington’s disease, Prion disease, Parkinson’s disease, Alzheimer’s disease and Spinocerebellar ataxia. Moreover, we found that the hub FRGs were also enriched in “focal adhesion”, “hippo signaling pathway”, “PI3K − AKT signaling pathway”, “carbon metabolism”, “oxidative phosphorylation”, “ubiquitin mediated proteolysis” and “proteasome”. Besides, we found that CD44 was closely related to virus infection including Coronavirus (also enriched in DDIT4), Epstein-Barr virus, Hepatitis B and Human papillomavirus. RB1 was related to many kinds of cancer such as prostate cancer, small cell lung cancer and hepatocellular carcinoma. CD44 and RB1 were related to the FoxO signaling pathway which is a reported regulator of cell senescence. Similarly, GSVA analysis suggested that overexpression of hub FRGs can induce AD by regulating various pathways and interactions (Figures 7F–J).

### 3.6 Immune cell infiltration analysis

**Figure 8:**
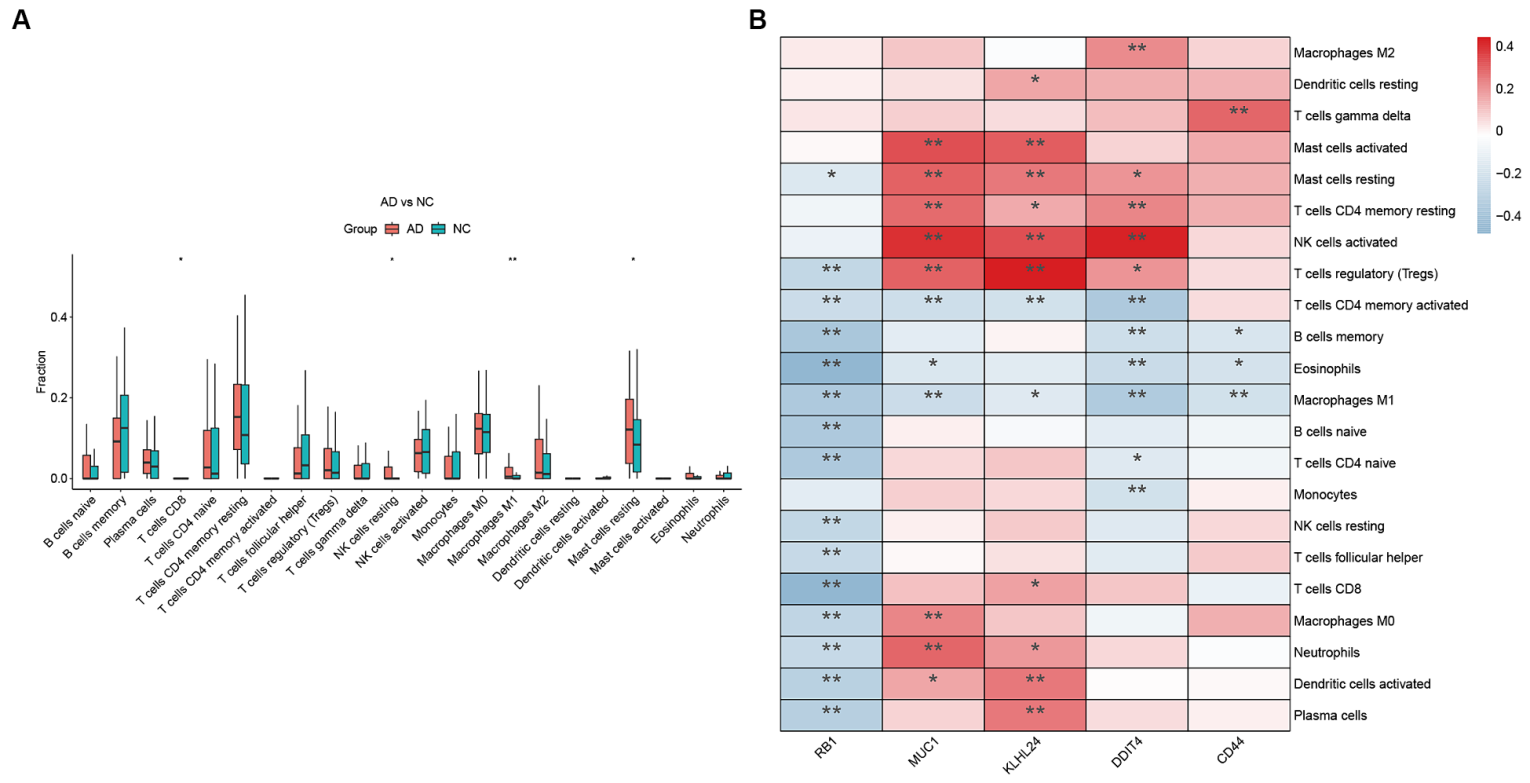
Immune cell infiltration analysis. **(A)** The CIBERSORT algorithm was utilized to investigate variances in the immune microenvironment between AD and healthy samples. **(B)** Pearson correlation analysis revealed that immune cells correlated with hub FRGs. (*p < 0.05 and **p < 0.01).

The immune microenvironment has been found to be closely linked to Alzheimer’s disease (AD). In order to investigate the differences in the immune microenvironments between AD patients and healthy samples, we utilized the CIBERSORT algorithm. Figure 8A demonstrates that AD samples have a higher proportion of resting natural killer (NK) cells, macrophages, and mast cells compared to healthy samples. Conversely, CD8 T cells were found to be expressed at lower levels in AD samples. Furthermore, we conducted a Pearson correlation analysis and discovered that regulatory T cells were positively correlated with the expression of MUC1, KLHL24, and DDIT4 (Figure 8B). Figure 8B illustrates that the expression of RB1 is negatively correlated with eosinophils and CD8 T cells. This suggests that RB1 may also play a role in modulating the immune microenvironment in AD. CD44 had strong positive and negative correlations with gamma delta T cells and macrophages, respectively. Overall, these findings provide evidence for the involvement of the immune system in AD pathogenesis and highlight the potential role of hub FRGs in shaping the immune microenvironment of AD patients.

### 3.7 Construction of the ceRNA network

**Figure 9:**
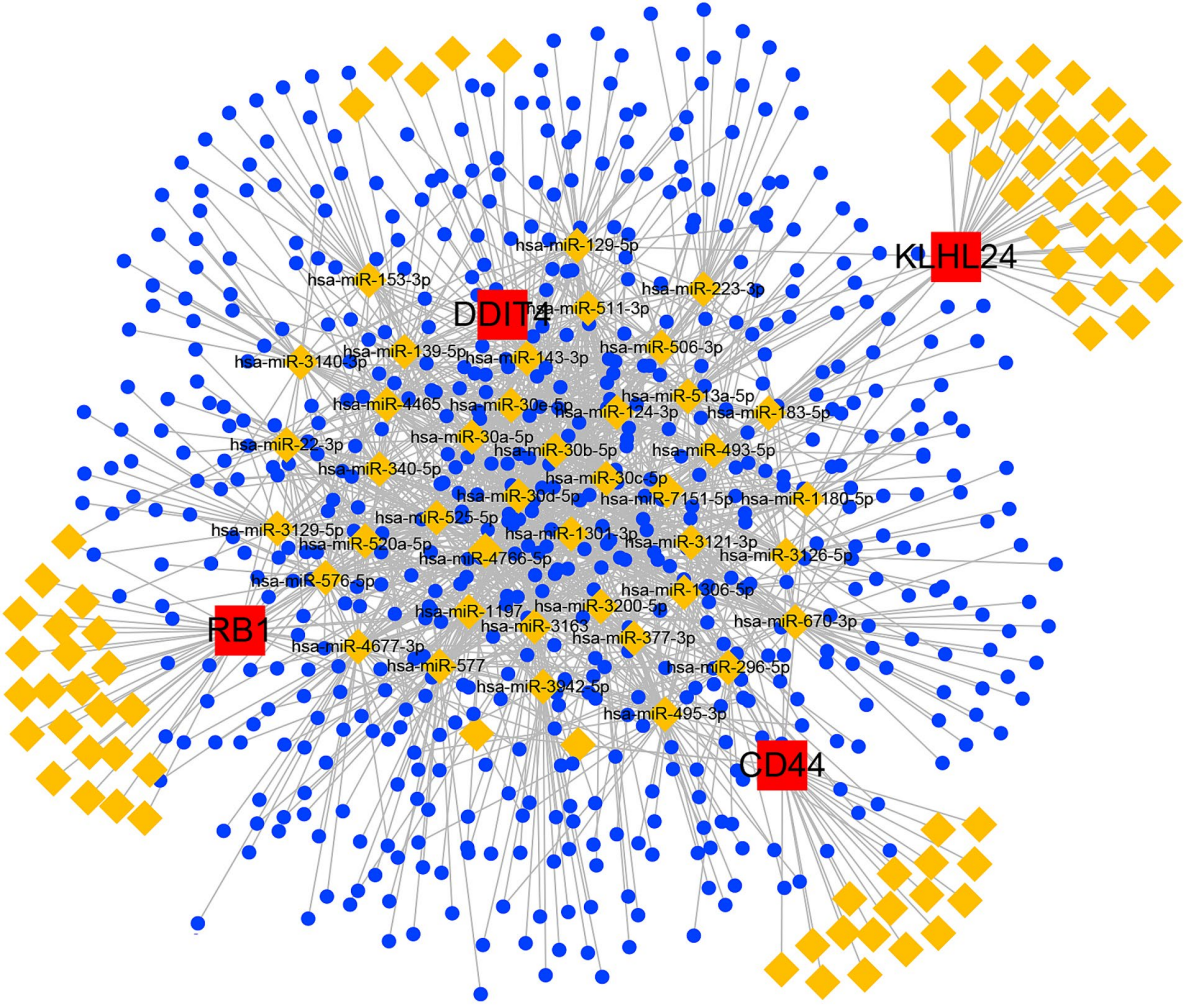
ceRNA networks based on hub FRGs.

Finally, we constructed a ceRNA network based on 5 hub FRGs through multiple non-coding RNA databases. The network included 746 nodes (4 hub FRGs, 123 miRNAs and 619 lncRNAs) and 1,601 edges (Figure 9). In detail, we found that a total of 241 lncRNAs could competitively bind hsa-miR-1180-5p, hsa-miR-143-3p, hsa-miR-296-5p, hsa-miR-3121-3p, hsa-miR-3126-5p, hsa-miR-4766-5p, hsa-miR-670-3p, hsa-miR-7151-5p and hsa-miR-513a-5p regulated CD44. For DDIT4, we found that 7 lncRNAs could regulate the expression of DDIT4 through competitive binding with 11 miRNAs. Among them, AL031282.2 was observed to competitively bind with hsa-miR-1306-5p, while AL391244.1 exhibited competitive binding with hsa-miR-30a-5p, hsa-miR-30c-5p, hsa-miR-30b-5p, hsa-miR-30e-5p, and hsa-miR-30d-5p. In the ceRNA network of KLHL24, there were 7 lncRNAs that could competitively bind 8 miRNAs and regulate the gene. Among these lncRNAs, AL139220.2 could competitively bind hsa-miR-124-3p and hsa-miR-506-3p. We identified a total of 12 lncRNAs that could competitively bind with 13 different miRNAs to influence the expression of the RB1 gene. Within this group, the shared lncRNA LINC01128 demonstrated competitive binding with hsa-miR-520a-5p and hsa-miR-525-5p. MUC1 was not found to be associated with regulatory non-coding RNAs through database searches. More details of the ceRNA network are shown in Supplementary Tables S2, S3.

## 4 Discussion

### 4.1 Conclusion

In this study, we conducted an analysis to investigate the role of certain genes related to ferroptosis in Alzheimer’s disease (AD). Specifically, we identified five genes (CD44, DDIT4, KLHL24, MUC1, and RB1) that are significantly associated with ferroptosis in AD, and are up-regulated in disease. Among these genes, DDIT4 and KLHL24 serve as markers for ferroptosis, while CD44, MUC1, and RB1 act as suppressors. To begin, we utilized bioinformatics to identify DEGs and DRGs in AD. Next, we compared these genes with the FerrDb database to identify FRGs in AD. We then performed GO and KEGG enrichment analysis to gain insights into the potential biological functions of FRGs. Additionally, using two machine learning algorithms, we identified hub FRGs. To further understand the gene function of these hub FRGs, we conducted single-gene GSEA and GSVA-KEGG analysis. We focused on the top 10 KEGG pathways to explore the involvement of hub FRGs in biological pathways. Furthermore, we utilized the CIBERSORT algorithm to investigate the relationship between immune cells and AD. We found that resting NK cells, macrophages, CD8 T cells, and mast cells were associated with the disease. This suggests that immune cells may play a role in the development of AD. Lastly, we constructed a ceRNA network to uncover non-coding RNA molecules that regulate the hub FRGs.

### 4.2 Overview of hub FRGs

In our study, we have found that hub FRGs play a crucial role in the development of AD and are up-regulated in disease. CD44 is a widely expressed cell surface adhesion molecule. The protein is a glycoprotein that spans the cell membrane and is composed of three domains: an extracellular domain, a transmembrane domain, and an intracellular domain (Chen et al., 2020). CD44 undergoes intramembrane proteolysis, resulting in the liberation of its intracellular domain and secretion of an Aβ-like peptide. There is evidence suggesting that Aβ-like peptides may contribute to the progression of Alzheimer’s disease by promoting neuroinflammation and neuronal loss (Lammich et al, 2002). Later research has demonstrated that splice variants of CD44, including CD44V3, CD44V6, and CD44V10, were significantly higher in AD patients compared to non-AD controls (Pinner et al., 2017). Additionally, increased CD44 gene expression was observed in lymphocytes taken from AD patients, with significantly increased levels of the unfolded p53 isoform expressed in the same cells. Correlations between p53 and CD44 expression have been established in cancer cells, suggesting that mutant p53 might be able to target CD44 as a gene, leading to the progression of AD via the regulation of neuronal ferroptosis (Uberti et al., 2010). Interestingly, CD44 is listed as one of the suppressors in the FerrDb database, and typically plays a negative regulatory role in biological processes. Several pieces of research have highlighted the role of ferroptosis in AD, with an increase in CD44 expression believed to be involved in the development of disease through this pathway (Bian et al., 2023; Ye et al., 2023). However, the precise mechanism of CD44 mediated AD by ferroptosis is still a controversial topic. We plan to investigate this further in our future work.

Our study investigated the protein DDIT4, also known as RTP801/REDD1, which is involved in regulating cellular growth, mitochondrial function, oxidative stress, and apoptosis (Pérez-Sisqués et al., 2021). Although, recent research has indicated that timely expression of REDD1 can limit energy consumption during metabolic stresses and prevent energy depletion, chronic expression of RTP801 has been linked to the pathogenesis of several diseases. Aβ-responsive genes were identified through cDNA microarray technology, and RTP801 emerged as a key regulator of Aβ toxicity. The results demonstrated that aberrant expression of RTP801 increased neuronal sensitivity to Aβ, leading to neuronal death, while down-regulation attenuated the cytotoxicity of Aβ (Kim et al., 2003). Further evidence came from a study demonstrating increased RTP801 levels in postmortem hippocampal samples from AD patients, with protein levels correlating with both Braak and Thal stages of the disease (Britto et al., 2020). Interestingly, the mouse model exhibited unexpected recovery of several gliosis hallmarks and key inflammasome proteins with neuronal downregulation of RTP801. These findings suggest that RTP801 may serve as a potential biomarker of neuroinflammation and neurotoxicity severity in Aβ-related neurodegenerative diseases, and may represent a viable target for AD prevention and treatment (Newcombe et al., 2018). Moreover, one study reported that hypoxic conditions and high cell density-induced DDIT4 expression are mediated by coactivation of molecules downstream in the PI3K/Akt signaling pathway. It is noteworthy that severe hypoxia can induce ferroptosis in cells (Jin et al., 2007). Thus, some molecules in the PI3K/Akt signaling pathway may activate ferroptosis, leading to the progression of AD.

The RB1 protein serves multiple functions in regulating the cell cycle, cell proliferation, and differentiation. It also acts as a crucial tumor suppressor gene, maintaining normal cellular function and inhibiting tumorigenesis (Dick et al., 2018). However, there have been limited direct studies linking RB1 to AD. One study reported an upregulation of mir-26b in AD, which has been shown to activate cell cycle entry, tau protein phosphorylation, and apoptosis in late mitotic neurons. The research further identified RB1 as a principal target that mediates the effects of miR-26b. Overexpression of miR-26b and inhibition of RB1 led to the activation of cyclin-dependent kinase 5 and an increase in tau phosphorylation at AD-relevant epitopes. This was followed by apoptosis and neurodegeneration in culture (Absalon et al., 2013). Additionally, RB1 may play a role in suppressing disease progression through ferroptosis, although the exact mechanism requires further investigation. In summary, while RB1 is primarily known for its involvement in cell cycle regulation and tumorigenesis, emerging research suggests its potential relevance in the context of AD. The upregulation of miR-26b and its impact on RB1 and downstream pathways provide insights into the role of RB1 in AD-related processes, such as tau phosphorylation and neuronal apoptosis. Further studies are needed to elucidate the exact mechanisms by which RB1 exerts its influence in AD.

### 4.3 Potential mechanism in AD

AD is characterized by a multifaceted pathogenesis encompassing a myriad of intricate biological processes and pathways. From the aggregation of amyloid-beta plaques to the hyperphosphorylation of tau proteins, the pathophysiology of AD unfolds through a cascade of events involving neuroinflammation, oxidative stress, synaptic dysfunction, and neuronal loss. These processes intertwine in a complex network, influencing each other and contributing to the progressive cognitive decline seen in AD patients. The results we obtained in our research may provide some new insights into the study of disease mechanisms. The results showed that autophagy and other pathways are involved in the progression of AD. Autophagy is a process of cellular self-degradation, through which cells are able to clear or recover damaged or excess components from within, in order to maintain the stability of the cellular environment (Li et al., 2023). One study suggested that autophagy reduces abnormal levels of β Starch-like protein deposition in the brain. LC3 (microtubule associated proteins 1A/1B light chain 3B) is a key molecule in the process of autophagy, playing an important role in the formation of autophagosomes and the regulation of autophagy. Researchers have found that the LC3/GABARAP family of proteins binds to the inner membrane through LC3 related endocytosis, assisting in the clearance of β Starch-like protein deposition and prevention of excessive inflammatory responses and neurodegeneration in the absence of microglia (Lipinski et al., 2010; Lee et al., 2023). Therefore, the imbalance of autophagy may lead to deposition of β-amyloid protein and thus, disease propagation. In addition, studies have found that glutathione, an antioxidant, is reduced in the AD brain when compared to healthy aging brains (Mandal et al., 2015). This also indirectly proves that brain oxidative stress and iron death are important pathological mechanisms in AD. Autophagy and ferroptosis are two mechanisms of cell death, and there is a certain mutual relationship and regulatory effect between them. But so far, no research has elucidated their regulatory relationship. Therefore, we deem it necessary to investigate the regulatory relationship between autophagy and ferroptosis in AD, in order to better understand the pathological processes involved in AD. This will be our next research direction.

### 4.4 Immune infiltration

The immune system is designed to defend us against external pathogens using an army of specialized cells. For a long time, the central nervous system was thought to be isolated from the rest of the body’s immune system by the blood brain barrier. Several important studies have revealed that T cells infiltrate different regions of the brains of individuals with AD and are positively associated with phosphorylated tau protein in two of these studies (van Olst et al., 2022). However, one study discovered decreased T cell numbers in the cortex and hippocampus of AD patients (Jevtic et al., 2017). Additionally, *ex vivo*-cultured human regulatory T cells are capable of modifying neuroinflammation in a preclinical model of AD (Faridar et al., 2022). These studies have been confirmed in part of our analysis. Our immunocyte infiltration analysis revealed that resting NK cells, M1 macrophages, and resting mast cells were highly expressed in the AD group, while CD8+ T-cells were lower compared to the healthy group. NK cells, M1 macrophages and mast cells in AD have not yet been reported. In addition, among different hub FRGs, MUC1 was found to be negatively associated with the activation of CD4 memory T cells. Although MUC1 is primarily known as an epithelial antigen, a study as early as 1998 suggested that it can also serve as an immunomodulatory molecule for human T lymphocytes (Agrawal et al., 1998). Further investigations have identified several peptide and glycopeptide epitopes of MUC1 that can bind to activated T cells and inhibit their proliferation (Agrawal and Longenecker, 2005). Additionally, MUC1 expression was found to be significantly upregulated in activated CD4 T cells (Konowalchuk and Agrawal, 2012). Whether the observed negative correlation between MUC1 and activated CD4 T cells in Alzheimer’s disease patients is valid, warrants further investigation. REDD1 is a stress response protein that is conserved across species and is known to be upregulated in response to a variety of cellular stressors including hypoxia, DNA damage, energy stress, ER stress and nutrient deprivation (Zhidkova et al., 2022). Our research has identified a positive correlation between DDIT4 expression and resting CD4 memory T cells, regulatory T cells, and CD8 T cells. Interestingly, previous studies have demonstrated that REDD1 is upregulated following T cell activation (Reuschel et al., 2015), suggesting that it may play a role in regulating the immune response. However, the relationship between T cell subtype and the expression of MUC1 remains unexplored, representing an exciting area for future investigation.

### 4.5 CeRNA network

Non-coding RNA refers to RNA molecules that do not encode proteins in cells, including miRNAs, lncRNAs, and circRNAs. Non-coding RNA regulates gene expression at the transcriptional and post-transcriptional levels in various diseases, serving as a biomarker and potential therapeutic target. An increasing number of studies have found that non-coding RNA is associated with pathogenic mechanisms in AD (Olufunmilayo and Holsinger, 2023). Our research found non-coding RNAs through the ceRNA network. With previous research, miR-143-3p is upregulated in the serum of AD patients, and inhibition of miR-143-3p promotes neuronal survival by targeting neuregulin-1 in an *in vitro* cell model (Sun et al., 2020). Additionally, hsa-miR-1306-5p in exosomes from serum has been shown to provide a protein-miRNA signature for differentiating between normal individuals, patients with mild cognitive impairment or vascular dementia, and sporadic AD patients in a pilot study (Li et al., 2020). The ubiquitous calpain-1, found in most tissues and organs, including the brain, is necessary for initiating synaptic plasticity and promoting neuroprotection. MiR-124-3p, a calpain-1 targeting miRNA previously reported to be downregulated in AD, functionally inhibited calpain-1 translation in a human neural cell line, HCN-2 (Zhou et al., 2019). In combination with these results, it appears that miR-143-3p, hsa-miR-124-3p and miR-1306-5p may serve as potential biomarkers for the diagnosis of AD. AL031282.2 and AL139220.2 may also be potential regulators impacting progression of AD.

### 4.6 Significance of the study

Through the application of bioinformatic methods, we have successfully identified five hub genes that connect iron metabolism and Alzheimer’s disease (AD). Our investigation also encompasses an exploration of the biological processes and pathways in which these genes are implicated, providing valuable insights into the development of AD. Furthermore, our findings indicate associations between these hub genes and various immune factors, pointing towards potentially significant roles within the immune microenvironment. Non-coding RNA molecules that target the hub FRGs could be utilized as promising indicators for the diagnosis of AD. In summary, this study offers a fresh perspective on understanding the pathological mechanisms involved in AD.

### 4.7 Limitation

One limitation of our study was the relatively small number of datasets. The restricted size of datasets constrained the depth and robustness of our findings. Furthermore, due to constraints in funding and human resources, we were unable to provide experimental data to support our hypotheses and conclusions. This lack of experimental data hindered the ability to validate our theoretical framework and explore causal relationships more comprehensively. Despite these limitations, we strived to maximize the utility of the existing data and employed rigorous analytical methods to draw meaningful insights within the confines of the available resources. We will address these limitations in future research by expanding the dataset size and ensuring sufficient resources for experimental validation, thereby improving the reliability and applicability of research results.

## Data availability statement

The datasets presented in this study can be found in online repositories. The names of the repository/repositories and accession number(s) can be found in the article/Supplementary material.

## Author contributions

YS: Writing – original draft, Writing – review & editing. QT: Writing – review & editing. WC: Writing – review & editing. LL: Writing – review & editing. YX: Writing – review & editing.

## Footnotes

^1^ https://www.ncbi.nlm.nih.gov/geo/

^2^ http://www.zhounan.org/ferrdb/current/

^3^ https://www.bioinformatics.com.cn/static/others/jvenn/example.html/
