# Supplementary material for: Genetic markers associated with ferroptosis in Alzheimer’s disease
Source: Front Aging Neurosci. 2024 Apr 22;16:1364605. doi: 10.3389/fnagi.2024.1364605 (PMC11073811; doi:10.3389/fnagi.2024.1364605)
Supplement: Supplementary file 1 [file Table_1.DOCX]

| Gene name | Expression | FeDb |
| --- | --- | --- |
| CD44 | UP | suppressor |
| DDIT4 | UP | marker |
| KLHL24 | UP | marker |
| MUC1 | UP | suppressor |
| RB1 | UP | suppressor |
